# Age-related methylation profiles of equine blood leukocytes in the *RNASEL* locus

**DOI:** 10.1007/s13353-015-0323-4

**Published:** 2015-11-09

**Authors:** T. Ząbek, E. Semik, T. Szmatoła, B. Oklejewicz, A. Fornal, M. Bugno-Poniewierska

**Affiliations:** 1 National Research Institute of Animal Production, Krakowska 1, 32-083 Balice, Poland; 2 Centre of Applied Biotechnology and Basic Sciences, University of Rzeszow, Sokołowska 26, 36-100 Kolbuszowa, Poland

**Keywords:** *RNASEL*, Horses, DNA methylation, Leukocytes, Aging

## Abstract

Methylation profiles across three CpG islands of the *RNASEL* gene were determined in blood leukocyte samples of Anglo-Arabian and Hucul horses. Bisulfite sequencing revealed hypomethylated state of the *RNASEL* promoter coinciding with methylated CpG island placed inside the gene. Several CpG sites were identified for which the methylation state was influenced by DNA polymorphism. Two of them showed monoallelic methylation. One of the CpG sites revealed functional polymorphism. A number of partially methylated CpG sites have been observed in the promoter area of *RNASEL*, which were used for the comparison of breed- and age-related effects. Clone bisulfite sequencing of blood leukocyte samples collected at different ages from particular individuals of AA and HC breeds and, also, BSPCR sequencing of 50 samples of juvenile and old AA and HC horses revealed increased methylation in particular CpG sites during aging. The age-related heterogeneity of white blood cells was hypothesized as being one of the potential causes of observed variability of methylation profiles in the *RNASEL* promoter.

**Electronic supplementary material:**

The online version of this article (doi:10.1007/s13353-015-0323-4) contains supplementary material, which is available to authorized users.

## Introduction

Aging encompasses a range of biological processes modified by different factors of environmental, genetic, and epigenetic nature (Bell et al. 2012). Studies concerning aging may be of interest in farm animal species. The possibility of determination of the biological age of farm animals can be important in terms of longevity, health, and extended age of reproduction (González-Recio 2011). A number of biochemical and genetic traits were involved in studying age-related processes of mammals. In addition, epigenetic marks including altered methylation profiles in conjunction with expression patterns have been widely described in the concern of cell senescence and aging in humans (Bell et al. 2012; Johansson et al. 2013; Day et al. 2013; Hannum et al. 2013). Methylation of DNA is a process catalyzed by methyltransferases and primarily affects CpG sites of mammalian genomes. This chemical modification has been widely mentioned in the concern of reduced activity of genes and silencing of foreign DNA (Hoelzer et al. 2008). It was recently shown that methylation patterns established at the initial stages of embryo development become deregulated with age during ontogenesis. These findings led to describing a new phenomenon called “age-associated epigenetic drift” (Johansson et al. 2013). Age-related methylation changes have been found to be important epigenetic traits for aging-related phenotypes (Bell et al. 2012). As an example, in the blood of humans, a number of age differential methylated regions (aDMRs) have been described to be associated with diseases of old age, including auto-immunity (Quintero-Ronderos and Montoya-Ortiz 2012). In this study, we assessed the impact of aging on the rate of methylation alterations of the *RNASEL* locus in the equine white blood cells. *RNASEL* belongs to the genes of the interferon signaling pathway. *RNASEL* encodes ribonuclease L, which, like other RNAses, is an important mediator for the activity of cytokines induced by the presence of viral RNA. Thereby, induced interferons activate 2′,5′-oligoisoadenylate synthetase, which, in turn, induces ribonuclease L, destroying all RNAs within the cell, including viral particles (Squire et al. 1994). It was found that the enzymatic activity of *RNASEL* increases in the early stages of life, reaching a maximum level in middle-aged adult animals, thereafter decreasing considerably with age (Pfeifer et al. 1993). RNase-L was found to mediate processes blocking cell proliferation and to suppress tumor progression in cancer. Because of this, its role was tested in cell senescence and longevity (Andersen et al. 2007). We have investigated age-related methylation changes of the *RNASEL* locus in blood leukocyte samples of Anglo-Arabian and Hucul horses. Both breeds substantially differ in conformation traits, types of utility, nutrition requirements, and healthiness. Different environmental determinants during the formation of Anglo-Arabians and Huculs might have led to the fixation of breed-specific epigenetic marks which we have also tried to identify on the DNA methylation level. Our aim was also to show the importance of genetic polymorphisms affecting the methylation state found in CpG islands.

## Materials and methods

### Samples

The study was done on two breeds of horses, Huculs and Anglo-Arabians. The DNA of white blood cells obtained from healthy horses was prepared and bisulfite converted using commercial kits. Two separate sets of samples were included in the range of bisulfite analysis.

#### Sample set A

First, blood samples derived from single individuals of both horse breeds were the subject of a cloned BSPCR sequencing approach. These included two blood samples of one Anglo-Arabian mare and one Hucul stallion. The mentioned blood samples were repeatedly collected at different ages of both individuals in order to resolve a couple of questionable parentage cases. Namely, the source material included blood samples of the Anglo-Arabian (AA) mare collected at 13 and 21 years of age and blood samples of the Hucul (HC) stallion were collected at age 21 months and 13 years. These four samples were used to pinpoint potential age-related differences unbiased by individual specificity of methylation patterns in the *RNASEL* promoter region.

#### Sample set B

The second set included 27 blood samples of AA horses (13 juvenile horses and 14 individuals aged between 16 and 21 years) and 23 blood samples of the HC breed (11 juvenile horses and 12 horses aged between 10 and 21 years). All of these samples were subject to the direct BSPCR sequencing approach in order to determine the methylation state (using an indirect method) of three CpG islands in the *RNASEL* locus and to refer to the results obtained using sample set A.

### BSPCR primer design and bisulfite amplification

CpG islands in the *RNASEL* locus were determined using recommendations implemented in Cpgplot software (Larsen et al. 1992). The predicted promoter regions of *RNASEL* were determined using MatInspector software (Cartharius et al. 2005). Bisulfite-PCR primers were designed in the regions free of CG sites using Methyl Primer Express® Software v1.0 (Life Technologies, Poland) (Table S1). Bisulfite DNA amplification (BSPCR) was performed with the use of HotStarTaq DNA Polymerase (Qiagen) and the two-step PCR protocol. The BSPCR reaction mixture contained: 10 ng template, 1× PCR buffer [Tris–HCl, KCl, (NH_4_)_2_SO_4_, pH 8.7], 2 mM MgCl_2_, 0.2 mM dNTP (Applied Biosystems, CA, USA), 0.125 μM primers, 1 U HotStar Taq polymerase [5 U/μL] (Qiagen, Poland) and an addition of Milli-Q water to a final volume of 20 μL. The BSPCR reaction conditions were as follows: initial denaturation at 95 °C for 15 min, 5 cycles of denaturation at 97 °C for 5 s, annealing for 2 min (specified in Table S1), elongation at 72 °C for 45 s, 35 cycles of denaturation at 97 °C for 5 s, annealing for 2 min (specified in Table S1), elongation at 72 °C for 45 s, and final elongation at 72 °C for 7 min.

### Direct BSPCR sequencing

BSPCR products of all individuals were sequenced using the BigDye® Terminator v3.1 Cycle Sequencing Kit (Life Technologies). The determined CpG islands were simultaneously searched for polymorphisms through sequencing of PCR products of unconverted DNA samples (Table S1). Sequencing reads of particular SNP variants were checked for putative amino acid changes of encoded protein using BlastX software (NCBI).

### Cloned BSPCR sequencing

Amplified fragments containing mixed methylated CpG sites were cloned using the TOPO TA Cloning Kit (Life Technologies, Invitrogen) and subjected to cycle sequencing with universal primers. The cloning procedure was done only for sample set A. Sequencing included 16 clones of 13 years old and 20 clones of 21 years old samples of the AA mare, and 19 clones of 21 months old and 18 clones of 13 years old samples of the HC stallion.

### Methods of methylation assessment using direct BSPCR or clone sequencing data and applied statistics

After the exclusion of DNA polymorphic sites, two types of data were investigated:

- Presence or absence of methylated cytosines in single CpG positions (simple pattern of methylation)
- Percentage of methylation (PM) of partially methylated cytosines in CpG sites

In the first case, the methylation status was assessed using direct BSPCR sequencing reads. In the second case, the percentage of methylation (PM) was determined based on cytosine counts at particular CpG positions using the sequencing data of cloned BSPCR products. The methylation level (PM values) was assessed and illustrated using BISMA software (Rohde et al. 2010). The variance of PM values were calculated to show the magnitude of methylation differences between samples which vary with age and breed (sample set A). In order to calculate the relative methylation percentage of partially methylated CpG sites in the 50 equine blood samples (sample set B), an indirect method proposed by Leakey et al. (2008) was applied (PM values in Table S2). A linear regression model was applied to assess the methylation differences at “fuzzy” methylated CpG sites (SAS package). Supporting Duncan’s tests were performed at the 0.05 level of significance.

## Results

### Methylation state of CpG islands

**Fig. 1:**
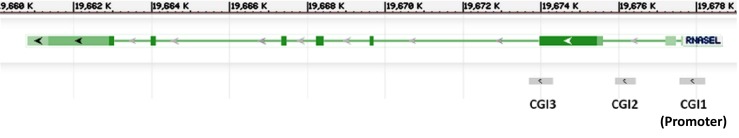
The layout of CpG islands (*grayed*) in the *RNASEL* locus based on the sequence alignment scheme (BLASTn). Exons are depicted as *green vertical bars* and the *arrows* indicate sequence orientation (gene annotated from the reverse strand of the genomic sequence)

Three predicted CpG islands (CGIs) of the *RNASEL* locus were the target of the study concerning methylation status and the effect of genetic polymorphisms in particular CpG sites. CGI1 was placed on the 5′ site covering the promoter region and the other two islands were placed inside the gene. CGI3 covered a part of exon 2 (Fig. 1). Bisulfite sequencing of 50 blood leukocyte samples revealed hypomethylated state of 18 CpG sites of CGI1 in opposite to hypermethylation detected in the CpG sites of islands located inside the gene (Table S3).

### DNA polymorphism affecting methylation state in the CG context

Alignment between the sequence of converted and unconverted DNA revealed three SNP-CpG sites in CGI1 and single sites in CGI2 and CGI3 in both horse breeds. The occurrence of T and G variants of the CGI1 region changed the methylation status of CpG9 and CpG21m respectively. In turn, the presence of allele A disrupted the methylation of single SNP-CpGs located in CGI2 and CGI3 (Table 1, Appendix S1). Pairwise comparison of epigenotype frequencies between SNP-CpG5 and CpG5 sites found in the CGI2 and CGI3 regions in both breeds revealed linkage disequilibrium between allele G and methylated cytosine within CpG sites (Table 1). In turn, the presence of allele A lead to the loss of methylated alleles at the mentioned CpG sites (example given in Appendix S1). The described CpG sites showed that monoallelic methylation occurred in both horse breeds with different frequencies (Table 1). The BlastX search revealed putative amino acid changes due to polymorphism at the SNP-CpG5 of the CGI3 region. The presence of allele A resulted in His to Arg non-conservative amino acid (aa) change at the 34 aa position of sequence no. ABN49949 (Table 1). Polymorphic CpG sites were excluded from investigations of age- and breed-related methylation changes.

**Table 1:** Epigenotype frequencies of CpG sites influenced by genetic polymorphism

Site	NCBI accession no.	Genomic location^a^	CGI	Breed	Epigenotype	Frequencies^b^
SNP-CpG9	ss#944501432	19677890	CGI1	AA	CC/CT/TT	0/0,069/0,931
				HC		0/0,167/0,833
SNP-CpG21	ss#944501434	19677716	CGI1	AA	CC/CG/GG	0,034/0,379/0,586
				HC		0,033/0,2/0,767
CpG5	–	19676068	CGI2	AA	CC/CT/TT	0,5/0,346/0,154 ^c^
				HC		**0,367/0,5/0,133** ^d^
SNP-CpG5	ss#944501428	19676069	CGI2	AA	GG/AG/AA	0,5/0,346/0,154
				HC		**0,367/0,5/0,133**
CpG5	–	19674196	CGI3	AA	CC/CT/TT	0,458/0,25/0,292
				HC		**0,208/0,583/0,208**
SNP-CpG5	ss#944501446^e^	19674197	CGI3	AA	GG/AG/AA	0,458/0,25/0,292
				HC		**0,208/0,583/0,208**

^a^ *Equus caballus* chromosome 5, EquCab2.0 (NCBI accession no. NC_009148)

^b^Epigenotype frequencies are ordered as homozygous for epiallele 1/heterozygous/homozygous for epiallele 2 (determined using BSPCR sequencing reads of 27 samples of AA and 23 samples of HC horses)

^c^Similar epigenotype frequencies between CpG5 and SNP-CpG5 found in the CGI2 and CGI3 regions among AA horses are underlined

^d^Similar epigenotype frequencies between CpG5 and SNP-CpG5 found in the CGI2 and CGI3 regions among HC horses are in **bold**

^e^Functional polymorphism resulting in 34 aa His to Arg substitution

### Methylation assessment in the concern of age and breed using clones and direct BSPCR sequencing data

Mixed methylation at the first eight CpG sites of CGI1 formed the basis for the calculation of methylation differences in the concern of breed and age. Comparison of PM values calculated from clone sequencing data and those based on the method of Leakey et al. (2008) showed similar results (Appendix S2). In silico search for transcription factor binding sites (TFs) covering sequences of partially methylated CpGs of the *RNASEL* promoter (Matinspector software) did not reveal any sites of TFs expressed in the blood cells.

#### Methylation assessment using sample set A

The sequencing results of cloned BSPCR products of leukocyte samples of different ages and breeds revealed similar averaged PM values across all CpG sites of the *RNASEL* promoter (Table S4). Concerning particular CpG sites, the greatest PM differences were observed for blood samples of male Hucul horses. The substantial age-related methylation decrease for CpG7 and significant methylation increase for CpG8 were detected in the Hucul blood samples (greatest values of variance) (Table S4). PM differences at both mentioned CpG sites between blood samples of different ages of the AA mare were proportional; however, they were substantially smaller (lower values of variance) (Table S4). In turn, large PM differences were detected in CpG7 between the blood samples of the 13-year-old male Hucul and the 13-year-old female AA horse (the greatest value of variance) (Table S5).

#### Methylation assessment using sample set B

Application of methylation assessment using the method of Leakey et al. (2008) did not reveal any significant differences in the concern of breed (Table 2). Implementation of the linear regression model for the data set comprising BSPCR sequencing reads of 50 blood samples confirmed the significant age-related changes at CpG8 and, additionally, revealed significant values for CpG4 and CpG5 (Table 2). The average methylation levels for CpG4, CpG5, and CpG8 were higher in a group of horses older than 1 year of age than those assessed for juvenile individuals in both breeds (Table 3).

**Table 2:** The effect of age and breed on eight CpG sites with the addition of distribution statistics

	GLM significance/distribution statistics								
	CpG1	CpG2	CpG3	CpG4	CpG5	CpG6	CpG7	CpG8	CpG average
Breed	ns	ns	ns	ns	ns	ns	ns	ns	ns
Age	.	ns	ns	**	***	ns	ns	***	***
*χ*	95.2	88.8	86.2	82.9	49.5	27.0	57.9	68.9	65.9
Median	94.6	89.5	87.9	83.5	50.9	28.9	58.3	71.6	66.1
SD	3.9	6.5	9.3	9.1	12.6	13.2	15.8	15.9	7.5

***p < 0.001; **p < 0.01; *p < 0.05; .*p* < 0.1; ns’ non-significant

**Table 3:** Mean percentage of methylation (PM) values of three CpG sites showing significant age-related differences of methylation profiles

Site	AA		HC		Both breeds	
	Juvenile	Adult	Juvenile	Adult	Juvenile	Adult
CpG4	70,78974^a^	76,05388	67,98497	77,79421	69,50422	76,85711
CpG5	77,18779	87,01256	80,2084	87,02527	78,57224	87,01843
CpG8	80,0514	85,22565	79,8284	83,69635	79,94919	84,51982

^a^PM was calculated using the method of Leakey et al. (2008) and averaged across samples of juvenile and adult horses within each breed separately and regardless of breed. Increased age-related hypermethylation is observed

## Discussion

### Description of methylation patterns across the *RNASEL* locus

Ongoing changes during ontogenesis affect the number of particular cell types, leading to modifications in tissue composition. One of the epigenetic features of these changes are alterations in DNA methylation patterns throughout the lifespan, which can be cell- and tissue-specific (Johansson et al. 2013; Jaffe and Irizarry 2014). This study describes the methylation patterns of the *RNASEL* locus in the equine blood leukocytes. Hypomethylation of the majority of CpG sites covering the *RNASEL* promoter region coincides with the hypermethylated state of CpG islands inside the gene. It was found that, in humans, the observed state of methylation patterns was a feature of about 70 % of genes actively transcribed throughout the lifespan (Day et al. 2013). In turn, a range of studies on human epigenomes allowed also to detect a number of CpG islands and single CpG sites with variable methylation levels during the lifespan (Bell et al. 2012; Johansson et al. 2013; Day et al. 2013; Hannum et al. 2013). In this study, a couple of “fuzzy” methylated CpG sites were observed in the *RNASEL* promoter, which were used to investigate some breed- and age-related changes.

### Polymorphic sites affecting methylation state in the CG context

Differences in methylation status affected by DNA polymorphisms were the source of interindividual epigenetic variation detected in both breeds. The presence of heterozygous genotypes in SNP-CpG sites overlapped with CpG monoallelic methylation, which occurred with different frequencies in particular breeds. Polymorphisms of SNP-CpG sites located in the promoter area could contribute to the binding effectiveness of methylation-dependent transcription factors. Such an impact of methylation on significant SNP-CpG sites has been described by Hellman and Chess (2010) and Shoemaker et al. (2010). In turn, one of the identified intragenic SNP-CpGs represents functional polymorphism, introducing non-conservative amino acid substitution in the region of the protein domain which would impair the enzymatic activity of *RNASEL*. It was found that impairment of *RNASEL* in old age results in increased susceptibility to viral infections in model animals (Pfeifer et al. 1993). Loss of methylation conditioned by the occurrence of allele A at this SNP-CpG site would, then, be a putative indicator of improper function of the enzyme.

### Age- and breed-related changes of methylation profiles in the *RNASEL* promoter

At first, partially methylated CpG sites of the *RNASEL* promoter were investigated using cloned BSPCR sequencing data. CpG hypermethylation was detected when comparing blood samples collected at different ages of the investigated individuals except for CpG7, where increased hypomethylation was observed. Thus, the obtained results confirmed the variation of methylation levels between CpG sites, even in short DNA stretches. The site-specific phenomenon of DNA methylation in the promoter area may be related to the dynamic nature of transcriptional machinery involving different chromatin states which determine the availability of particular transcription factor binding sites (Marchal and Miotto 2015). It should be noted that white blood cells include a number of leukocyte subtypes which can reveal different states of methylation in particular genomic areas (Talens et al. 2010). Because of this, it is possible that observed differences of methylation between blood samples collected from the AA mare and HC stallion at different ages might coincide with changes in the cellular fraction of blood leukocytes during aging. In addition, observed age-related methylation could overlap with breed- or sex-specific effects (when comparing CpG7 methylation profiles between blood samples of the HC male and AA female of similar age). For example, detailed evidence for sex and ethnic specificity of age-related methylation profiles has been given using microarrays methylome data of white blood cells of the healthy human population (Hannum et al. 2013). To assess the magnitude of methylation changes at partially methylated CpG sites in the *RNASEL* promoter area, we also applied an indirect measure of PM values (Leakey et al. 2008) across a set of 50 blood leukocyte samples from both horse breeds. A general tendency observed in the studied equine leukocyte samples was consistent with the age-related methylation increase of particular CpGs located in promoter regions (Johansson et al. 2013). It is interesting that the use of the indirect method of methylation assessment did not reveal any significant breed-specific effects across eight CpG sites of the *RNASEL* promoter. The sole source of differences between both horse breeds were genetic polymorphisms affecting the methylation state of CpG sites. The only result being common to both methods of methylation assessment in this study (cloning procedure versus the indirect measure of PM values) is the occurrence of increased methylation during age. Hypermethylation of CpG8 in the *RNASEL* promoter could, then, be treated as a potential age-related epigenetic feature of equine white blood cells. A further question is if the detected “fuzzy” methylated CpG sites in the promoter area are of functional importance for *RNASEL* activity.

The conducted research revealed the presence of locus-specific methylation changes which have potential value for studies on aging of the blood system of the horse. The inclusion of genetic and epigenetic variation into association and functional studies may provide novel findings about *RNASEL* activity during viral infections.

## Electronic supplementary material

Below are the links to the electronic supplementary material.

Appendix S1 (PDF 89 kb)

Appendix S2 (PDF 15 kb)

Table S1 (PDF 255 kb)

Table S2 (XLSX 15 kb)

Table S3 (XLSX 11 kb)

Table S4 (XLSX 10 kb)

Table S5 (XLSX 9 kb)
