# Supplementary material for: Age-related methylation profiles of equine blood leukocytes in the RNASEL locus
Source: J Appl Genet. 2015 Nov 9;57:383–8. doi: 10.1007/s13353-015-0323-4 (PMC4963465; doi:10.1007/s13353-015-0323-4)
Supplement: Supplementary file 1 — (PDF 89 kb) [file 13353_2015_323_MOESM1_ESM.pdf]

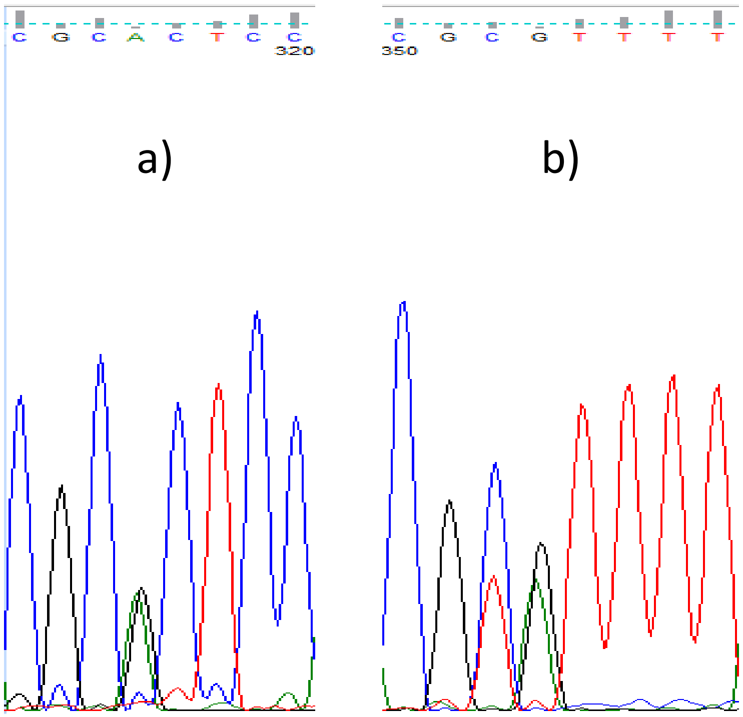

Appendix S1 Monoallelic methylation state of CpG5 influenced by A/G polymorphism (NCBI ss#944501428) found in CGI2

a - sequence of unconverted DNA; b - sequence of DNA after conversion; Cytosine of one of the alleles followed by adenine is converted to thymine while cytosine of the second allele followed by guanine is methylated
