# Supplementary material for: Age-related methylation profiles of equine blood leukocytes in the RNASEL locus
Source: J Appl Genet. 2015 Nov 9;57:383–8. doi: 10.1007/s13353-015-0323-4 (PMC4963465; doi:10.1007/s13353-015-0323-4)
Supplement: Supplementary file 2 — (PDF 15 kb) [file 13353_2015_323_MOESM2_ESM.pdf]

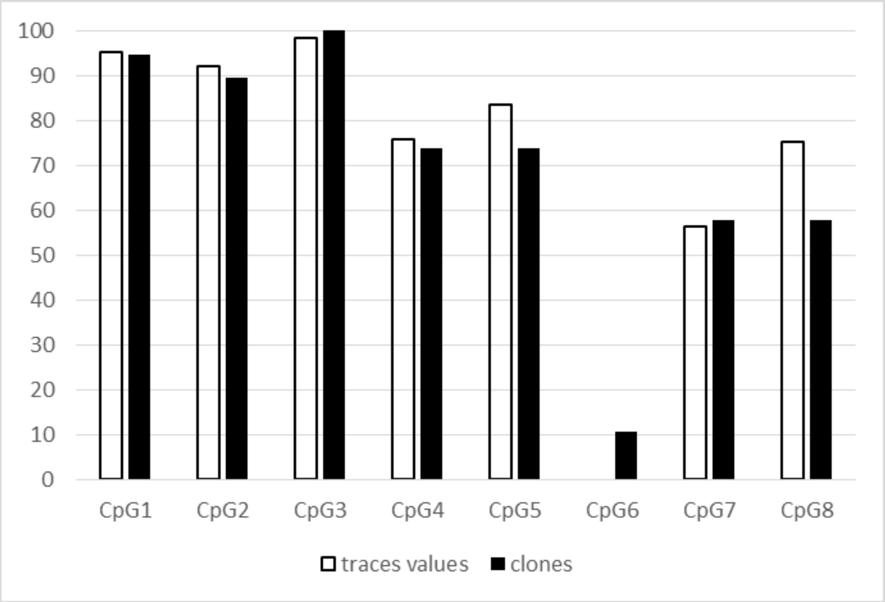

Appendix S2 Comparison of PM values generated using direct BS-PCR (traces values) and the clone sequencing approach (blood leukocytes from 21 month old hucul male).
