# Supplementary material for: Age-related methylation profiles of equine blood leukocytes in the RNASEL locus
Source: J Appl Genet. 2015 Nov 9;57:383–8. doi: 10.1007/s13353-015-0323-4 (PMC4963465; doi:10.1007/s13353-015-0323-4)
Supplement: Supplementary file 3 — (PDF 255 kb) [file 13353_2015_323_MOESM3_ESM.pdf]

Table S1 Primers for amplification of bisulfite converted (BS PCR) and unconverted DNA (PCR) designed using NCBI sequence No. NC_009148.

RNASEL CpG island	Start	Stop	CG %	BSPCR oligos (5'-3')	Start	Stop	Size (bp)	Ta (°C)	PCR oligos (5'-3')	Start	Stop	Size (bp)	Ta (°C)
CGI1	19677577	19678210	52.8	F:tggctgcaggaccagatctccagg	19678187	19678210	634	62/60	F:tgcaggaccagatctccag	19678188	19678206	625	59
				R:agagagctggttctttcagggat	19677577	19677599			R:tgaaagaaccagctctcttcct	19677582	19677603		
CGI2	19675849	19676447	39.1	F:tagatttgagaatcctaaaattgatc	19676422	19676447	599	54/52	F:gctgtgaccagtcaagtttttg	19676396	19676417	502	59
				R:tcaggtgccaatctttgaaaaaag	19675849	19675872			R:ttgctttgtcttcccaaacc	19675916	19675935		
CGI3	19673700	19674295	46.8	F:ctggggttctatgaggagcaa	19673700	19673721	596	54/52	F:ctggggttctatgaggagca	19674276	19674295	594	59
				R:atgaagccatgtcctggatgga	19674275	19674295			R:catccaggacatggcttcat	19673702	19673721		
